# Lemierre’s Syndrome in the 21st Century: A Literature Review

**DOI:** 10.7759/cureus.43685

**Published:** 2023-08-18

**Authors:** Abhinav Tiwari

**Affiliations:** 1 Department of Surgery, University Hospital Southampton NHS Foundation Trust, Southampton, GBR

**Keywords:** lemierre’s syndrome, sepsis, thrombophlebitis, fusobacterium, tonsillitis, pharyngitis, microbiology, haematology, otolaryngology

## Abstract

Lemierre’s syndrome is a rare, life-threatening complication of an acute oropharyngeal infection. It is generally characterised by pharyngitis secondary to *Fusobacterium necrophorum*, causing thrombophlebitis of the internal jugular vein and sepsis, with subsequent formation of septic emboli that can rapidly spread to different organ sites. The condition is associated with high mortality if treatment with antibiotics is delayed, and recent evidence suggests that patients are at significant risk of in-hospital morbidity and long-term neurological sequelae. Although it is agreed that antibiotics are the mainstay of treatment, there is currently no consensus on the use of anticoagulation in the condition. This review article aims to summarise our current understanding of Lemierre’s syndrome with regard to its definition, epidemiology, microbiology, presentation, diagnosis, and treatment.

## Introduction and background

Lemierre’s syndrome is a rare, life-threatening complication of an acute oropharyngeal infection. The syndrome is generally characterised by a pharyngeal infection that invades the parapharyngeal space, causing thrombophlebitis of the internal jugular vein (IJV) and sepsis [1]. This leads to the formation of septic emboli that can rapidly spread to many different organ sites, including the lungs, joints, brain, and liver. Antibiotics are essential in the treatment of Lemierre’s syndrome, and the condition was associated with high mortality before their advent [2]. The use of therapeutic anticoagulants in Lemierre’s syndrome is well reported, though evidence to support their proposed benefits is currently lacking [1]. This review article aims to provide a summary and discussion of our current understanding of Lemierre’s syndrome in the 21st century.

## Review

Definition

Although there is general agreement on the key characteristics of Lemierre’s syndrome, there is currently no consensus on its definition. In 1936, Dr André Lemierre, a professor of bacteriology in Paris, published a case series of 20 patients who developed septicaemias due to anaerobic organisms [3]. This paper lay the groundwork for establishing the characteristic clinical features of the condition that would later go on to be named Lemierre’s syndrome. Lemierre reported that the primary infection could originate from various sites, including the pharynx, mouth, jaw, ear, and gastrointestinal and genitourinary tracts. He noted the association with anaerobic organisms, in particular, *Bacillus funduliformis* (the current synonym for *Fusobacterium necrophorum*), but he noted that other anaerobes such as *Bacteroides fragilis* and *F. nucleatum* may also be implicated. Lemierre noted two other important features, namely, the presence of local thrombophlebitis following the primary infection, and the presence of metastatic septic lesions, stating that “septicaemias observed by myself have never been pure septicaemias, they have always been accompanied by the formation of distant metastatic abscesses” [3].

Lemierre’s original work established the key clinical features of this eponymous syndrome. However, the lack of a clear definition in his paper has likely contributed to the differing inclusion criteria that have been used by subsequent authors. Some require the primary infection to originate in the oropharynx [4,5], whereas some broaden this to any infection in the head and neck region [6]. Lemierre himself stated that the abdomen and pelvis may also be a source of primary infection, though some authors choose to omit such cases [3,7]. One proposed explanation for this discrepancy may be the demographic differences among patients according to the site of their primary infection [7]. Lemierre’s syndrome originating in the oropharynx is more typically seen in young adults [4], otomastoiditis as a primary source is more common among toddlers and children [7,8], and patients with *F. necrophorum* infections originating outside of the head and neck region are usually middle-aged [8]. Authors also differ in their microbiological criteria; some require a positive blood culture with any organism [5], some require the growth of any *Fusobacterium* species [9], and some require *F. necrophorum* specifically [4]. Some definitions require the presence of radiologically proven jugular vein thrombosis [10], though others use evidence of metastatic septic emboli as an alternative to this criterion [4]. Some authors have also further classified Lemierre’s syndrome as typical and atypical [9]. One suggested criterion has been a combination of clinical, microbiological, and radiological criteria, including (i) a history suggestive of oropharyngeal infection in the last four weeks, (ii) evidence of metastatic lesions to end organs, and (iii) evidence of IJV thrombophlebitis or isolation of *F. necrophorum* from blood cultures [2]. Clearly, no consensus has been reached, and this represents a potential area of future work that will help to promote collaboration and pooling of data between centres.

Epidemiology

Lemierre’s syndrome was relatively common before the advent of antibiotics [2]. Its incidence fell after the introduction of antibiotics for oropharyngeal infections [11], leading to it being termed the “forgotten disease” in the 1990s [12]. There has been a reported rise in its incidence in the last 30 years [11], with up to 5.5 cases per million being reported in 2017 [8]. The reasons for this rise are unclear. Several explanations have been proposed including a restriction on the use of antibiotics for sore throats by primary care physicians, increasing antibiotic resistance, and fewer tonsillectomies [2,13]. Better identification of the condition may also play a role, with the improvement of anaerobic blood culture techniques and increased use of high-resolution imaging for the detection of IJV thrombophlebitis. [7,13].

Lemierre’s syndrome typically affects healthy young adults. A systematic review in 2021 that included 712 patients with the condition reported that the median age was 21 (interquartile range = 17-33) [9]. While less common, there are also reports that have described its occurrence in both children and the elderly [14,15]. Several studies have also reported a slight male preponderance, though there seems to be no adequate explanation for this [16,17]. As reported by Riordan in 2007, it is difficult to know if there is a clear ethnic predisposition to the condition, though in his review he noted cases from Europe, North America, South America, and Asia [2]. Finally, the mortality associated with Lemierre’s syndrome was high in the pre-antibiotic era, with Lemierre himself reporting that 90% of patients in his original case series died [3]. Although reported rates in the 21st century are much lower at around 2-4%, it is important to note that this may rise if antibiotics are delayed [8,9,18].

Causative agent

The causative agent in Lemierre’s syndrome is typically *F. necrophorum*, an anaerobic Gram-negative bacillus that can be found within the normal flora of the upper respiratory tract [7]. Studies have found that *F. necrophorum* may be present in the throat swabs of up to 21% of healthy individuals [19,20], and may also be the second-most common pathogen isolated from the throat swabs of patients with sore throats [21]. The role of *F. necrophorum* in recurrent tonsillitis has also been suggested, with one study reporting that patients with recurrent sore throat had a significantly higher load of *F. necrophorum* in their throat swabs compared to patients with acute sore throats and healthy controls [20].

The mechanism by which *F. necrophorum* causes Lemierre’s syndrome is unclear. There is evidence to suggest that the organism lacks important genes that are required for invasion [22]. As such, one hypothesis is of co-infection, where a primary invading pathogen causes local mucosal damage and inflammation which assists in establishing an infection with *F. necrophorum* [23]. The Epstein-Barr virus (EBV) has been suggested as one of these organisms and has been reported previously [24-26], though it is not seen in all cases of the condition [27]. The true association between the two organisms remains to be seen. Once established, it is hypothesised that *F. necrophorum* may spread to the IJV via the tonsillar vein, via the lymphatic system, or by the direct invasion and attachment of peritonsillar abscesses onto veins found in the loose connective tissue of the pharynx [1,2].

It is not currently clear whether *F. necrophorum* is the only organism responsible for causing Lemierre’s syndrome. Although some authors require its presence as part of the condition’s definition, studies that omit this criterion have found that the organism is not isolated in all cases [9]. Furthermore, there are reported cases of the condition where cultures have isolated other anaerobic organisms such as *F. nucleatum* and *Bacteroides fragilis* [28,29]. Taken together, this would suggest that other organisms may well be implicated in causing Lemierre’s syndrome. However, *F. necrophorum* not being isolated in all reported cases may in part be due to false negatives, due to both antibiotics having been started before cultures, and the long incubation period associated with culturing anaerobic organisms [7]. This has been highlighted in cases of Lemierre’s syndrome where molecular methods such as polymerase chain reaction have proven the presence of *F. necrophorum* despite cultures failing to isolate the organism [30]. The presence of other organisms in cultures can also be explained by the co-infection hypothesis [23], and this is supported by the fact that polymicrobial cultures are not uncommon in cases of Lemierre’s syndrome [8]. As such, it is still possible that *F. necrophorum* may be the sole causative agent responsible for causing the condition, but there is currently not enough evidence to draw a definitive conclusion.

Presentation and complications

In his initial case series, Lemierre stated “the appearance and repetition several days after the onset of a sore-throat (and particularly of a tonsillar abscess) of severe pyrexial attacks with an initial rigor, or still more certainly the occurrence of pulmonary infarcts and arthritic manifestations, constitute a syndrome so characteristic that mistake is almost impossible” [3]. This continues to be a robust description of this eponymous syndrome and touches on many of its important clinical features.

The signs and symptoms of Lemierre’s syndrome generally appear in two stages. The first stage is the primary infection. As Lemierre described, this is usually an acute pharyngitis that manifests as a sore throat [3]. This may also be accompanied by other non-specific features of infection such as fever [8]. Some cases of untreated pharyngitis may progress to a peritonsillar abscess manifesting as a severe unilateral sore throat with odynophagia [31,32]. A recent systematic review found that 73% had experienced signs or symptoms of a recent acute oropharyngeal infection, though other sites of infection including the ear, lower respiratory tract, and neck were also reported [9]. Other primary infections such as cellulitis [33], perianal abscess [34], and pericoronitis have also been previously reported within the literature [35].

The second stage of Lemierre’s syndrome is of IJV thrombophlebitis and sepsis, which usually occurs several days after the primary infection [8]. Patients typically present to hospital acutely unwell with sepsis or septic shock [8,16]. It is important to note that the primary infection may already have resolved, and sepsis or its sequelae may mask the symptoms of the primary infection [1], a phenomenon that can make early diagnosis challenging. IJV thrombophlebitis may manifest with symptoms of ipsilateral neck pain and headache and clinical signs of swelling along the sternocleidomastoid muscle [36]. These features are not seen among all patients. A review in 2009 that included 114 patients with Lemierre’s syndrome found that 20% reported neck pain and only 23% had a neck mass on examination [13]. Similarly, a prospective series of 37 patients in Denmark with oropharyngeal Lemierre’s syndrome found that 63% had unilateral swelling of the neck on admission [16]. The authors reported that this was often mistaken as lymphadenitis or peritonsillar abscess, perhaps reflecting the rarity of IJV thrombosis more generally.

Patients with Lemierre’s syndrome may often present to the hospital with evidence of metastatic lesions to end organs [8,9]. When septic IJV thrombophlebitis occurs, the septic emboli formed can spread to many different sites, including the lungs, joints, brain, and liver [9]. This can cause significant end-organ pathology that may warrant the need for surgical intervention. The lungs are usually the affected organ, and patients may go on to develop pneumonia, empyemas, lung abscesses, pleural effusions, or septic pulmonary emboli [4,16]. These typically manifest with symptoms such as pleuritic chest pain, shortness of breath, cough, and haemoptysis [37]. Lemierre himself noted the pulmonary manifestations being announced by “intense thoracic pain of sudden onset, by dyspnoea, sometimes by blood-stained or rusty sputum, by pleural frictions, and by localised areas of subcrepitant rales” [3]. The joints are usually the second most common site for the spread of septic emboli and are affected in around 5-15% of cases [4,9]. This typically manifests as septic arthritis which presents with pain in the affected joint [3]. A review by Riordan in 2007 found that the hip, knee, and shoulder were among the commonly affected joints [2]. Septic arthritis may also lead to osteomyelitis of the adjacent bone in some cases [38]. Although rare, septic emboli can spread to the central nervous system, and this is associated with significant morbidity and mortality [9,13]. Central nervous system spread can result in several complications, including meningitis, encephalitis, cavernous sinus thrombosis, cerebral abscess, and cerebral infarction [1]. Septic emboli can rarely metastasise to other sites and result in a liver abscess [39], splenic abscess [40], pericarditis [41], iliopsoas abscess [42], soft tissue abscess [43], and ophthalmological complications [44].

Finally, although the mortality associated with Lemierre’s syndrome is much lower than it was in the pre-antibiotic era [3,8], evidence from a recent systematic review suggests that hospitalised patients may continue to be at significant risk of in-hospital morbidity, including new venous thromboembolism and new peripheral septic lesions. Furthermore, up to 10% of survivors may go on to develop long-term sequelae, including cranial nerve palsy, blindness, reduced visual acuity, paralysis, paresis, and functional limitations [9]. All treating clinicians should be aware of this potential for serious morbidity, and clinical vigilance should be maintained throughout the patient’s hospital stay.

Diagnosis

Lemierre’s syndrome can be challenging to diagnose in its early stages. An eight-year national retrospective study in Sweden found that of 104 patients with Lemierre’s syndrome, 59% had previously attended for a related healthcare visit, and effective antibiotics were given to less than 10% of these patients [8]. These findings are probably a reflection of the fact that Lemierre’s syndrome is virtually indistinguishable in its early stages from other more common differentials of a sore throat such as viral tonsillitis, glandular fever, and influenza. The condition’s rarity may also play a role; doctors may not have learned about the disease in medical school and may not have seen it in clinical practice. The early recognition of Lemierre’s syndrome arising from sites other than the oropharynx may prove to be even more challenging, and a high index of suspicion is thus paramount.

Patients typically present to the hospital acutely unwell with sepsis, though clues to diagnosis at this stage may continue to be sparse [8,16]. This is because sepsis may mask the initial symptoms of the primary infection and specific examination findings, for example, signs of IJV thrombophlebitis, may not always be present [1,13,16]. Blood tests will typically show raised inflammatory markers and may show thrombocytopenia [8]. EBV serology may also be carried out as part of the diagnostic workup. Cultures usually play a key role, and the isolation of *F. necrophorum* from blood cultures may be the first clue to the diagnosis [45]. However, blood cultures may take weeks to reveal anaerobic organisms, and these may be negative if empirical antibiotics were started before cultures [7]. As such, the use of cultures cannot necessarily be relied upon, as patients may have already developed metastatic septic lesions by the time the results come back. The use of throat swabs in primary care may be one method to flag a suspicion of Lemierre’s syndrome at an earlier stage. However, this technique is limited by the fact that most throat swabs are cultured aerobically, and distinguishing *F. necrophorum* from the throat’s normal flora can be difficult, even when swabs are cultured anaerobically [46].

Imaging usually helps to reach a diagnosis and there are pros and cons to each technique. CT scan with contrast is generally considered the gold standard because it is readily available and because of its ability to visualise both IJV thrombosis and metastatic lesions to end organs [1,47]. Doppler ultrasound is a radiation-free technique that can also be used to visualise thrombosis, though it is less sensitive than CT [47,48]. MRI scanning may be the most reliable imaging technique, though its use is limited by cost and availability [49,50]. One review of 137 cases of Lemierre’s syndrome found that CT was used to visualise IJV thrombophlebitis in 95% of cases, ultrasound was used in 5%, and MRI was not used at all [50]. Finally, other imaging tools may be used as adjuncts, for example, plain film chest radiographs for those with suspected pulmonary manifestations [13].

Treatment

The management of Lemierre’s syndrome requires a multidisciplinary team approach [44]. Several teams including microbiologists, otolaryngologists, radiologists, intensivists, haematologists, and medical specialists may be involved. Although care should be individualised, the general principles of management remain. Antibiotics are the mainstay of treatment with beta-lactams or carbapenems in combination with metronidazole being commonly prescribed [50]. *F. necrophorum* is almost always sensitive to metronidazole, co-amoxiclav, clindamycin, and imipenem, and less likely to be sensitive to erythromycin and penicillin [51]. If there is any diagnostic doubt, timely therapy with intravenous broad-spectrum antibiotics should be commenced and further refinement should occur based on the results of available sensitivities. There is no consensus on the optimal duration of antibiotic treatment and studies have reported the average length of treatment to be around three to five weeks [16,52]. Some authors suggest at least two weeks of intravenous antibiotics before considering a switch to oral therapy [52]. Alongside antibiotics, patients with Lemierre’s syndrome may also require surgical intervention as part of the management of metastatic septic lesions. This may include procedures such as IJV ligation, pleural drainage, abscess drainage, mastoidectomy, tonsillectomy, and invasive cranial surgery [8,9]. Such patients should be promptly referred to the appropriate surgical team.

The use of therapeutic anticoagulation has been reported in around 23-56% of cases of Lemierre’s syndrome [2,9]. Various anticoagulants have been used including low-molecular-weight heparin, fondaparinux, unfractionated heparin, direct oral anticoagulants, and vitamin K antagonists [9]. The reported duration of treatment ranges between 70 and 84 days [9,52]. The hypothesis for the use of anticoagulation is that bacteria may be concealed within the thrombus, so treatment to stop its progression may increase the accessibility of antibiotics to the source of infection, leading to faster resolution of the disease [52]. Some argue instead that as the thrombus is secondary to an infectious process, the resolution of the infection with antibiotics would by itself cause the thrombus to resolve [52]. Others suggest that the use of anticoagulation may in fact propagate the spread of septic emboli to end organs [53].

The current evidence is mixed. An observational study of 82 patients with Lemierre’s syndrome in Sweden found no significant difference in outcomes, including thrombosis progression, peripheral septic complications, and 30-day mortality, between patients who were given treatment-dose anticoagulation, prophylactic-dose anticoagulation, and no anticoagulation [4]. Similarly, a single-centre, 16-year, retrospective case series of 18 patients with Lemierre’s syndrome in the United States found no difference in thrombosis outcomes between those who did and did not receive anticoagulation [10]. However, both studies had small sample sizes and as such may have been statistically underpowered to reveal any true effect.

A recent systematic review that included 712 patients with Lemierre’s syndrome between 2000 and 2017 found that 14.3% developed new venous thromboembolism or peripheral septic lesions during hospitalisation [9]. The study reported that these thromboembolic events were less frequent in patients who received anticoagulation. Furthermore, the use of anticoagulation was found to be independently associated with a lower risk of combined new thromboembolic or septic complications in a multivariable model. Additionally, the use of anticoagulation did not seem to be associated with a risk of major bleeding events in the analysis. Although this data would support the hypothesis that anticoagulation may be of benefit, caution should be used in the interpretation of these results, as the study mainly consisted of retrospective case reports and small case series that may have been subject to selection bias. Finally, some advocate for the use of anticoagulation in select cases of Lemierre’s syndrome, describing its use in rarer complications, for example, cavernous sinus thrombosis [54]. No consensus has been reached and further prospective data is needed to be able to draw conclusions and make any recommendations for the use of anticoagulation in Lemierre’s syndrome.

Future work

Much of the current evidence on Lemierre’s syndrome comes from retrospective, single-centre case reports or small case series. A recent systematic review by Valerio et al. that included Lemierre’s syndrome cases published between 2000 and 2017 found that of 540 studies identified, 480 were single case reports, 48 were case series of up to five patients, and only two case series included more than 20 patients [9]. While this is probably explained by the condition’s rarity, other factors including the lack of a standardised definition and core outcome set may also play a role. Nevertheless, these studies are likely prone to selection, reporting, and publication bias, and the lack of high-quality evidence means that associations between risk factors and outcomes, for example, the use of anticoagulation on thromboembolic events, can only be hypothesised. Although the condition’s low incidence makes conducting randomised controlled trials virtually unfeasible, further prospective evidence may help to address these issues. An ongoing area of work directly related to this is the Bacteria-Associated Thrombosis, Thrombophlebitis and LEmierre syndrome (BATTLE) registry [55], which is an ambispective, multi-centre, multi-disciplinary registry that was set up in 2021 and aims to address the limitations of current evidence, guide management, and improve outcomes of patients affected by Lemierre’s syndrome. Results from datasets such as this may help to progress our understanding of this complex condition in the future.

## Conclusions

All clinicians should consider Lemierre’s syndrome as a differential in patients presenting with sepsis following a sore throat. The condition is a life-threatening complication of acute pharyngitis that can lead to the formation of septic emboli through IJV thrombophlebitis. Although rare, its incidence may be on the rise. There is currently no standardised definition of the condition within the literature, which may in part contribute to its low-quality evidence base. Early diagnosis of Lemierre’s syndrome is challenging due to its non-specific nature. Patients will typically present to the hospital acutely unwell with sepsis and some may present with metastatic septic lesions to end organs. Recent evidence suggests that patients are at significant risk of short and long-term morbidity. CT scan with contrast is generally considered gold-standard for diagnosis. *F. necrophorum* is the classical causative agent, though there is currently not enough evidence to conclude that it is the sole organism responsible for causing the condition. Prompt treatment with antibiotics is essential to reduce mortality risk, and surgical intervention may be required in some patients. Additional management with anticoagulants is well reported, though much of the current data comes from retrospective, single-centre studies. More prospective evidence is needed to make any recommendations on the use of anticoagulation in Lemierre’s syndrome.
